# Exogenous GABA-Ca Alleviates Growth Inhibition Induced by a Low-P Environment in Peanuts (*Arachis hypogaea*)

**DOI:** 10.3390/antiox13111414

**Published:** 2024-11-18

**Authors:** Zhiyu Sun, Mingzhu Ma, Huan Liu, Dongbing Tao, Shaikh Amjad Salam, Xiaori Han, Yifei Liu, Jean Wan Hong Yong

**Affiliations:** 1 China-Australia Joint Laboratory for Plant Nutrition and Germplasm Resources Innovation, College of Land and Environment, National Engineering Research Center for Efficient Utilization of Soil and Fertilizer Resources, Northeast China Plant Nutrition and Fertilization Scientific Observation and Research Center for Ministry of Agriculture and Rural Affairs, Shenyang Agricultural University, Shenyang 110866, China; 2 College of Food Science, Shenyang Agricultural University, Shenyang 110866, China; 3 Institute of Agriculture, The University of Western Australia, Perth, WA 6009, Australia; 4 School of Biological Sciences, The University of Western Australia, Perth, WA 6009, Australia; 5 Department of Biosystems and Technology, Swedish University of Agricultural Sciences, 23456 Alnarp, Sweden

**Keywords:** gamma-aminobutyric acid, calcium, signal crosstalk effect, p deficiency, cyclic electron flow, ROS toxicity

## Abstract

Phosphorus (P) deficiency is a major global factor constraining peanut production. Exogenous γ-aminobutyric acid (GABA) and Ca^2+^ are essential to improve stress resilience in peanuts growing under low-P conditions. This study therefore examined the detailed physiological effects of GABA-Ca on restoring peanut growth under low-P conditions. These included the root–shoot ratio, leaf nutrients, photochemical activity, reactive oxygen species (ROS), cyclic electron flow (CEF), ATP synthase activity, and the proton gradient (∆pH), all of which were measured under low-P (LP, 0.5 mM) and optimized-P (1 mM) conditions. Specifically, supplying GABA-Ca under LP conditions regulated the ∆pH by causing adjustments in CEF and ATP synthase activities, buffering the photosystems’ activities, restoring the antioxidant enzyme system, and lowering ROS production. Interestingly, exogenous GABA-Ca restored peanut growth under low-P conditions, possibly by the putative signaling crosstalk between GABA and Ca^2+^. The plausible signal amplification between GABA and Ca^2+^ suggested that the combination of GABA and Ca, may offer an effective strategy for enhancing peanut adaptation to low-P conditions. Moving forward, the strategic supplementation of GABA-Ca, either during cultivation or through the formulation of novel fertilizers, opens up many possibilities for better and more resilient plant production in soils with low P.

## 1. Introduction

Phosphorus (P) is a critical major limiting nutrient for plant growth; in particular, its availability is restricted in tropical regions, with a resulting impact on global food production [1,2]. Varying degrees of P limitation are common in terrestrial ecosystems, and the extent of phosphorus limitation varies in different climate zones and regions [1]. P has low mobility and a high fixation rate in soil [2,3] resulting in less than 25% of phosphatic fertilizer being utilized during the growing period. The high reliance of conventional and often intensive farming activities on phosphatic fertilizers is inevitable for maintaining high production in order to cope with increasing food demands [4,5]. The long-term and excessive use of phosphatic fertilizer has caused negative environmental issues such as the eutrophication of water bodies and poor drinking water quality. In China, about 131 major lakes are classified as highly eutrophic [6]. Furthermore, the intense and unsustainable use of phosphatic rocks could deplete the finite resource of naturally occurring phosphatic rocks by 2050 [7,8]. Moreover, the effective management of phosphate-based fertilizers to improve agricultural productivity while maintaining environmental sustainability is indeed a very challenging task globally. Specifically for China, a country with a large population, the efficient management of P resources, P utilization, sustainable food production, and food security requires strategic, science-based, multi-disciplinary, and multi-stakeholder policies [9].

P is an essential element for plant growth and production [10,11]. It also regulates nucleic acid, protein, ATP, and various P-containing enzymes in plants. It is involved in important physiological and biochemical processes in plants [10,11]. Even a slight P deficiency will lower photosynthesis and cause growth inhibition in peanuts [12]. Low P triggers ROS accumulation and damages the photosynthetic membranes of peanut plants [13]. Additionally, an increase in the ∆pH of peanut thylakoids under low-P stress induces changes in photosynthetic control and strengthens the non-photochemical quenching (NPQ) photoprotection mechanism, along with cyclic electron flow (CEF) around PSI.

Peanut is an important oilseed and leguminous crop that is highly sensitive to variation in P availability in soils [14]. In China, and globally, peanut production in low-yielding fields is often associated with cultivation in P-limiting environments [15]. Peanuts are physiologically sensitive to the availability of P, and minor phosphorus deficiency will lower photosynthesis and increase ROS toxicity, ultimately reducing growth [12,13]. As low-P-induced ROS stress is linked to photosynthetic perturbations, efforts to reduce ROS and enhance photosynthesis are crucial for restoring peanut production in P-deficient soils.

GABA (γ-aminobutyric acid) is well known and proven for physiological improvement, particularly for mental and physical relief. Because of its non-toxic and health-beneficial properties, it is widely used in the food industry as a natural edible additive [16,17,18,19]. In recent years, GABA has been gradually applied in agricultural production due to the large-scale industrial production of bio-based green pathways [20,21,22]. Supplemental GABA accumulates rapidly in plants and enhances their resistance to abiotic stress [21]. A stress-induced increase in GABA synthesis also leads to changes in the intracellular calcium flux [23]. Glutamate decarboxylase (GAD), a key enzyme involved in the GABA shunt pathway, was identified as a calmodulin (CaM)-binding protein that can be stimulated by Ca^2+^ and CaM [24]. Stress in plants generally elevates the levels of Ca^2+^ and GABA simultaneously; thus, the application of both supplements may offer an avenue to help plants adapt to unfavorable conditions [25]. Based on the literature, Ca^2+^ is an important second messenger in plants [26,27]. Under abiotic stress, increasing the levels of free Ca^2+^ in the cytoplasm and binding with calmodulin will initiate various physiological and biochemical processes [26,28]. GABA is known to be involved in regulating plant metabolism through signaling molecules, especially during unfavorable conditions [21]. Ma et al. [29] found that the interplay of Ca^2+^ and GABA improved the stress resilience of barley, particularly during exposures to high NaCl levels. Interestingly, Ca^2+^ signaling is involved in the accumulation of GABA under stress; GABA also transmits Ca^2+^ signaling through receptors to trigger downstream signaling cascades [30]. There is plausible evidence of a complex system of crosstalk between GABA and Ca^2+^ signaling. Xie et al. [31] also suggested that utilization of GABA and Ca^2+^ interactions possibly enhanced the resilience of soybeans to high NaCl levels.

From earlier studies, GABA effectively alleviated the growth inhibition and photoinhibition of peanuts grown under low-P stress [13]. Exogenous applied Ca^2+^ treatments were shown to enhance the photosynthesis of peanuts under P-limited environments [15]. However, it remains unclear whether the combination of GABA and Ca can deliver positive effects on enhancing peanuts’ resilience to low P. Thus, this study aimed to evaluate the physiological and biochemical effects of using Ca^2+^, GABA, and GABA-Ca treatments on peanuts, measured under low-P (LP, 0.5 mM) and optimized-P (1 mM) conditions.

## 2. Materials and Methods

The Chinese peanut (*Arachis hypogaea* L.) cultivar “Xiaobaisha” was used in the study. Uniformly sized peanut seeds were selected and transplanted into 60 pots (11.4 cm height, 13.6 cm diameter, 1 seed per pot), each containing an equal amount of sand. Each treatment had 12 pots of peanut plants to provide sufficient biological materials for the different analyses, and there were 3 independent biological replicates for each measurement. Peanut seeds were cultured in an incubator (Conviron, Winnipeg, MB, Canada). Peanuts were grown on a 12/12 h photoperiod (light intensity, 1000 μmol quanta m^−2^ s^−1^), 28/23 °C day/night temperature, and 60% relative air humidity. Prior to treatment, Hoagland’s nutrient solution was applied every 3 days. After 21 days of planting, pure water was added to flush away the local residual nutrients and salt patches from the pots.

The pots with uniformly sized peanut seedlings were then divided into five treatments: (1) control plants (CK) (normal P level + foliar spray of ultrapure water); (2) LP (low P level + foliar spray of type 1 ultrapure water); (3) LP + Ca (low P level + foliar spray of 15 mM CaCl_2_); (4) LP + G (low P level + foliar spray of 10 mM GABA); and (5) LP + GCa (low-P stress + foliar spray of GABA-Ca solution contained 10 mM GABA and 15 mM CaCl_2_). Hoagland’s nutrient solution was prepared in accordance with the previous studies [12,13]. Starting 27 days after planting, the leaves of the peanut plants were sprayed twice a day (at 8:00 and 16:00) for three days (at 27, 28, and 29 days after planting) according to different treatments. With reference to Hoagland’s nutrient solution, it was determined that the low-P-concentration solution (−P, 0.5 mM) corresponded approximately to having 15.5 mg/kg ± 3 mg/kg of soil available P, typically found in soils of medium-yielding to low-yielding peanut fields; the normal-P-level solution (CK, 1 mM) was equivalent to that of high-yielding peanut fields with soil available phosphorus exceeding 30 mg/kg.

Two types of Hoagland’s nutrient solution were prepared in the form of KH_2_PO_4_ for simulating normal CK plants (1 mM) and low P for LP plants (0.5 mM) 22 days after planting. The levels of nitrogen (N) and potassium (K) in the two P levels of Hoagland’s nutrient solution were balanced by altering the amounts of KNO_3_ and NH_4_NO_3_. Every two days, 25 mL of the nutrient solution for each treatment was supplied.

At 30 days after planting, chlorophyll fluorescence and P700 parameters were measured on the third youngest fully expanded leaf of peanut plants using a Dual-PAM 100 (Heinz Walz, Effeltrich, Germany) according to the method of [12,26]. The light intensity gradient setting of the RLCs was set to 0, 22, 48, 71, 144, 226, 284, 513, 771, 1190, 1467, and 1821 μmol quanta·m^−2^·s^−1^. The CEF was determined by the formula CEF = ETR (I) − ERT (II), and the ratio of the quantum yield of CEF was Y (II) [Y (CEF)/Y (II)] = [Y (I) − Y (II)]/Y (II) [13,26]. ΔpH and membrane potential (ΔΨ) were measured simultaneously using a Dual-PAM 100 with a P515/535 module, following the method of [13,26].

The net photosynthetic rate (Pn) was monitored on the third youngest fully expanded leaf with an open gas exchange system (GFS 3000; Heinz Walz, Effeltrich, Germany) at 30 days after planting. The setting parameters of the instrument program were chosen with reference to the previous method [12,13]. Relative chlorophyll concentration was determined with a chlorophyll meter (SPAD-502 plus, Tokyo, Japan) at 31 days after planting.

At 32 days after planting, the superoxide radical (O_2_^•−^) generation rate, leaf hydrogen peroxide (H_2_O_2_) concentration, and MDA concentration of peanut leaves were measured. Malondialdehyde (MDA) in peanut leaves was assayed following the method of Heath and Packer [32,33] and our modified version for peanuts [13]. Peanut leaf samples (0.2 g) frozen in liquid nitrogen were homogenized with trichloroacetic acid and centrifuged. The absorbance of thiobarbituric acid-reactive substances in the supernatant was measured at 450, 532, and 600 nm for the calculation of MDA content. H_2_O_2_ in peanut leaves was assayed following the method of [13] with modifications. Peanut leaf samples (0.2 g) frozen in liquid nitrogen were homogenized with acetone and centrifuged. The supernatant was mixed with 5% TiCl4 and 2 mol H_2_SO_4_ and centrifuged at 3000 r/min for 10 min. The supernatant was taken, and its absorbance at 415 nm was measured; the H_2_O_2_ content was then calculated. The generation rate of O_2_^•−^ was assayed following the method of [34,35] and the modified version of Sun et al. [13]. Peanut leaf samples (0.2 g) frozen in liquid nitrogen were homogenized with 50 mM phosphate buffer (PBS, pH 7.8) and centrifuged at 10,000 r/min for 20 min at 4 °C. The supernatant was mixed with 1 mM hydroxylamine hydrochloride and stored at 25 °C for 60 min. The mixture was supplemented with 17 mM p-aminobenzene sulfonic acid and 7 mM 1-naphthylamine and stored at 25 °C for 20 min. The absorbance of the mixture at 530 nm was measured, and the O_2_^•−^ content (μmol/L) was calculated.

Antioxidant enzymes—superoxide dismutase (SOD), peroxidase (POD), ascorbate peroxidase (APX), and catalase (CAT)—were extracted and their activity assayed according to the previous methods [36,37,38,39]. Peanut leaf samples (0.5 g) frozen in liquid nitrogen were homogenized with 50 mM PBS (pH 7.8) and centrifuged at 10,000 r/min for 20 min at 4 °C. The supernatant was taken and mixed with PBS containing nitroblue tetrazolium, EDTA-Na_2_, methionine, and riboflavin. After light treatment, its absorbance at 560 nm was measured, and the SOD activity was calculated. Peanut leaf samples (0.5 g) frozen in liquid nitrogen were homogenized with 200 mM PBS (pH 6.0) and centrifuged at 10,000 r/min for 20 min at 4 °C. The supernatant was taken and mixed with PBS containing guaiacol and H_2_O_2_. Its absorbance at 470 nm was measured at 1 min intervals for a total of 3 min. The POD activity was calculated based on the change in absorbance values. Peanut leaf samples (0.5 g) frozen in liquid nitrogen were homogenized with 0.15 M PBS (pH 7.8) and centrifuged at 10,000 r/min for 20 min at 4 °C. The supernatant was taken and mixed with PBS containing H_2_O_2_. The absorbances at 240 nm were measured at 1 min intervals for a total of 4 min. The CAT activity was calculated based on the decrease in absorbance values. Peanut leaf samples (0.5 g) frozen in liquid nitrogen were homogenized with 0.05 M PBS (pH 7.0) and centrifuged at 10,000 r/min for 20 min at 4 °C. The supernatant was taken and mixed with PBS containing EDTA-Na_2_, PBS containing ascorbic acid, and PBS containing H_2_O_2_. Its absorbance at 290 nm was measured over a period of 40 s, and the APX activity was calculated.

At 33 days after planting, leaf area and plant height were measured. Peanut leaves (0.2 g DW) were digested with a mixture of H_2_SO_4_ and H_2_O_2_. Leaf N concentrations were determined using the micro-Kjeldahl method [40]. Leaf P concentrations were determined using the vanadomolybdate method [41]. The mixture contained peanut leaves (0.1 g DW), 5 mL HNO_3_ (65%, guaranteed reagent), 2 mL H_2_O (type 1 ultrapure water), and 1 mL H_2_O_2_ (30%, guaranteed reagent); the leaves in the mixture were digested with a microwave system (Milestone Ethos one, Italy). Program settings followed previous studies [13]. The amounts of potassium (K), calcium (Ca), magnesium (Mg), iron (Fe), manganese (Mn), copper (Cu), and zinc (Zn) were determined by flame atomic absorption spectrometry (AA-4002400304, PerkinElmer, Waltham, MA, USA). Ca, Mg, Fe, Mn, Cu, and Zn content were obtained as metal concentrations and DW Values.

The experiment adopted a random block design. The results presented the mean values and standard errors of three biological replicates of a final experiment. A one-way ANOVA model including a homogeneity-of-variance test and Fisher’s LSD test in Origin 2024 was used for statistical analysis. The results were presented as mean values and standard errors of three biological replicates. Different letters above columns in the figures indicate significant differences among the variants (*p* ≤ 0.05). All graphs were plotted using GraphPad Prism 9.5 software.

## 3. Results

### 3.1. Growth

**Figure 1:**
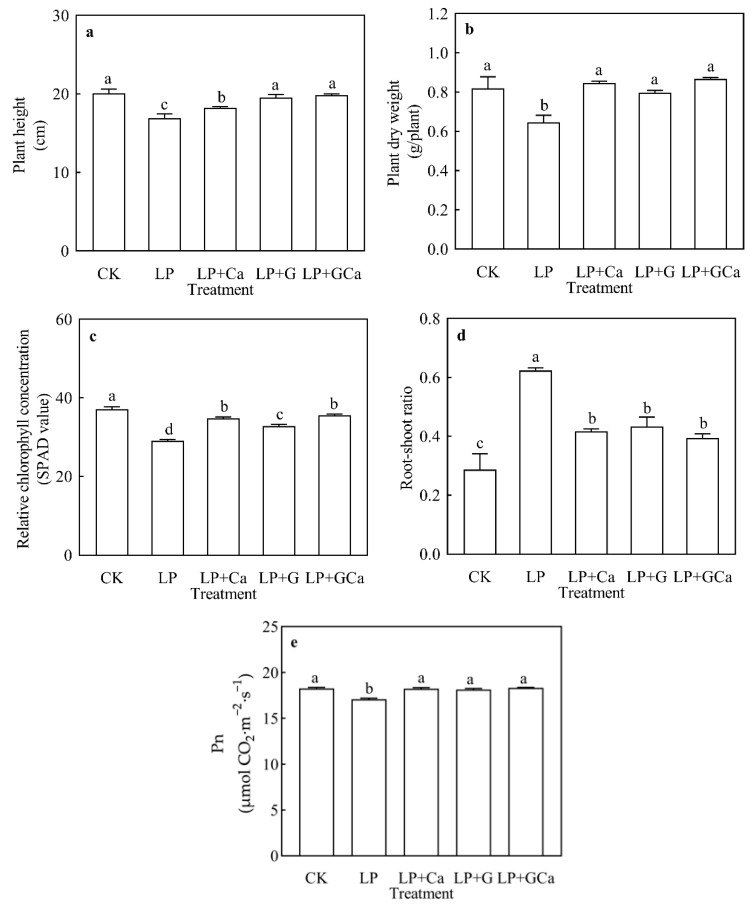
Effects of exogenous GABA-Ca on growth and development in peanuts under low-P stress. Plant height (**a**), Plant dry weight (**b**), Relative chlorophyll concentration (**c**), Root-shoot ratio (**d**), and Pn (**e**). Values are means of three biological replicates ± SE (*n* = 3). Different and unshared letters above columns in the figures indicate significant differences among the 5 variants (*p* ≤ 0.05).

The peanut plants’ growth and physiological parameters were tested through the utilization of GABA and calcium supplementation. Compared with CK, plants that had received LP treatment showed lower plant height, plant dry weight, SPAD value, and Pn (Figure 1a–c,e). The treatment LP + GCa enhanced the plant height, plant dry weight, SPAD value, and Pn compared with LP. LP treatment significantly increased the root–shoot ratio of the peanut (Figure 1d), and LP + GCa significantly decreased the root–shoot ratio (Figure 1c). The LP + G group showed greater plant height than the LP + Ca group; the LP + Ca group showed greater plant dry weight and SPAD values than the LP + G group (Figure 1a–c).

### 3.2. Mineral Element

**Figure 2:**
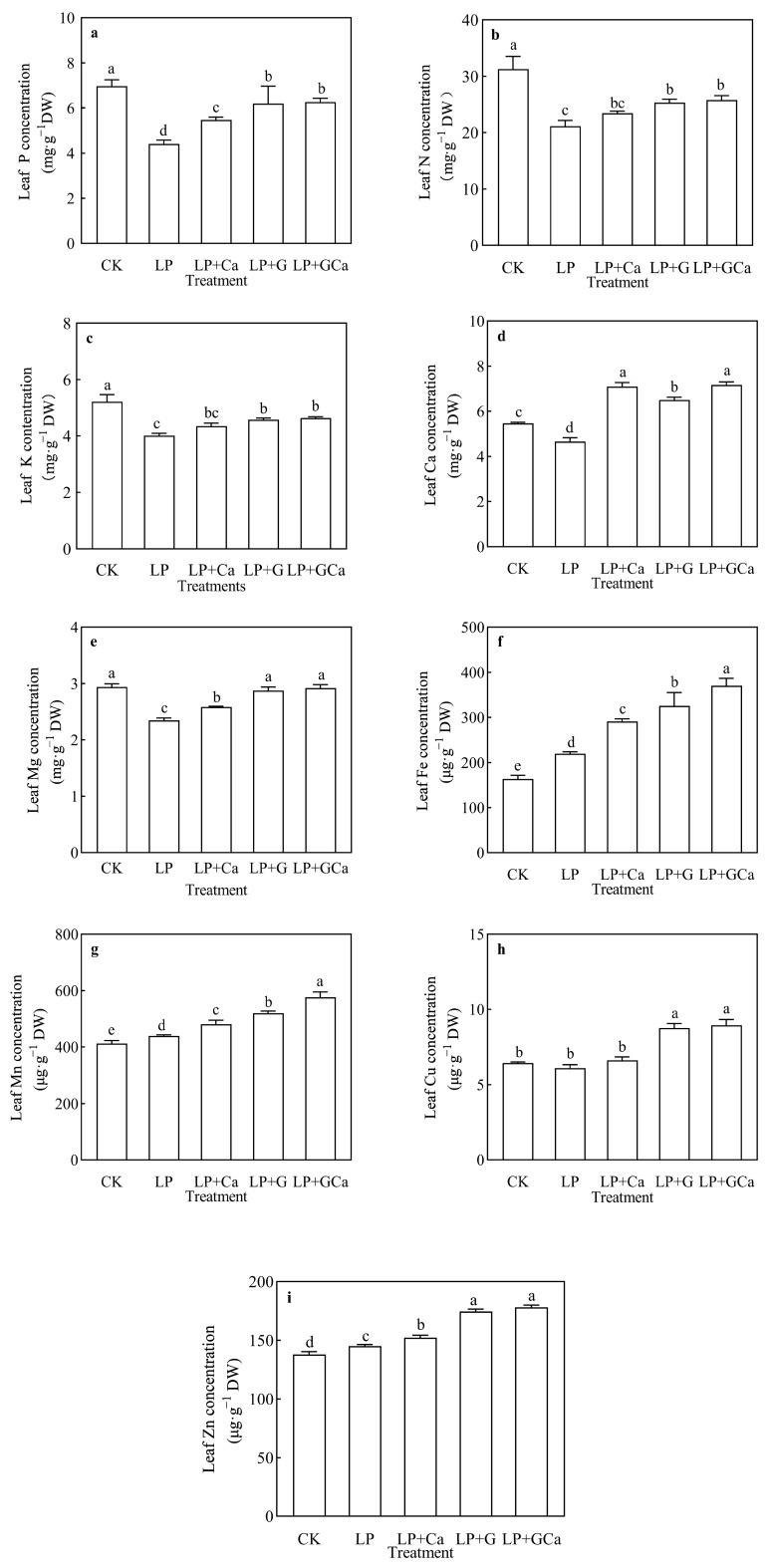
Effects of exogenous GABA-Ca on P (**a**), N (**b**), K (**c**), Ca (**d**), Mg (**e**), Fe (**f**), Mn (**g**), Cu (**h**), and Zn (**i**) levels in peanut leaves under low-P stress. Values are means of three biological replicates ± SE (*n* = 3). There are different and unshared letters above columns in the figures which indicate significant differences among the 5 variants (*p* ≤ 0.05).

Compared with CK, LP treatment resulted in a lower P concentration in leaves. According to the obtained results, LP + G and LP + GCa significantly increased the observed nutrient levels in peanut plants. LP + G treatment showed higher P concentration in leaves than LP + Ca (Figure 2a). LP + G and LP + GCa treatments significantly increased the N concentration of peanut leaves, LP + Ca, but the effect of the applied treatment was not obvious (Figure 2b).

### 3.3. ROS

**Figure 3:**
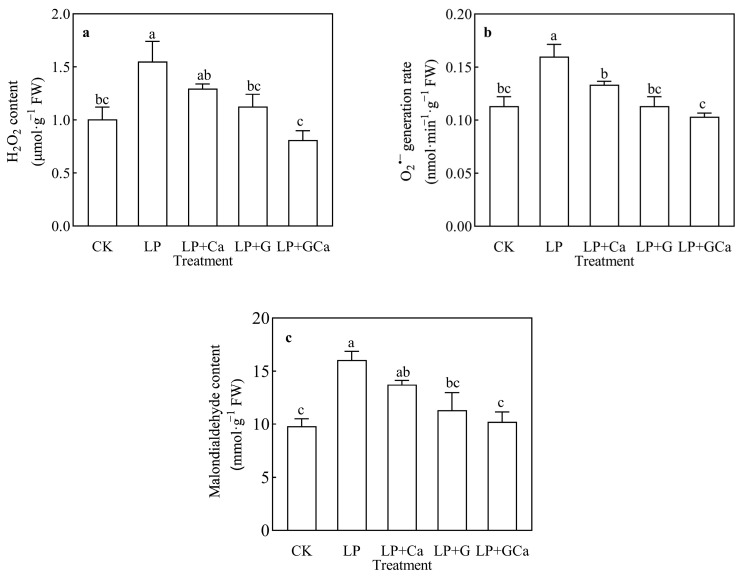
Effects of exogenous GABA-Ca on H_2_O_2_ content (**a**), O_2_^•−^ generation rate (**b**), and MDA content (**c**) in peanut leaves under low-P stress. Values are means of three biological replicates ± SE (*n* = 3). Different and unshared letters above columns in the figures indicate significant differences among the 5 variants (*p* ≤ 0.05).

The LP treatment caused a higher leaf O_2_^•−^ generation rate and H_2_O_2_ level compared with CK (Figure 3a,b). Furthermore, exogenous GABA-Ca alleviated oxidative damage in peanut leaves under LP stress, as indicated by a significant reduction in MDA content (Figure 3c). LP + Ca, LP + G, and LP + GCa treatments significantly reduced the ROS in peanut leaves under low P. The combined application of GABA and Ca proved more effective than the sole application of GABA or Ca.

### 3.4. Antioxidant Enzyme Activity

**Figure 4:**
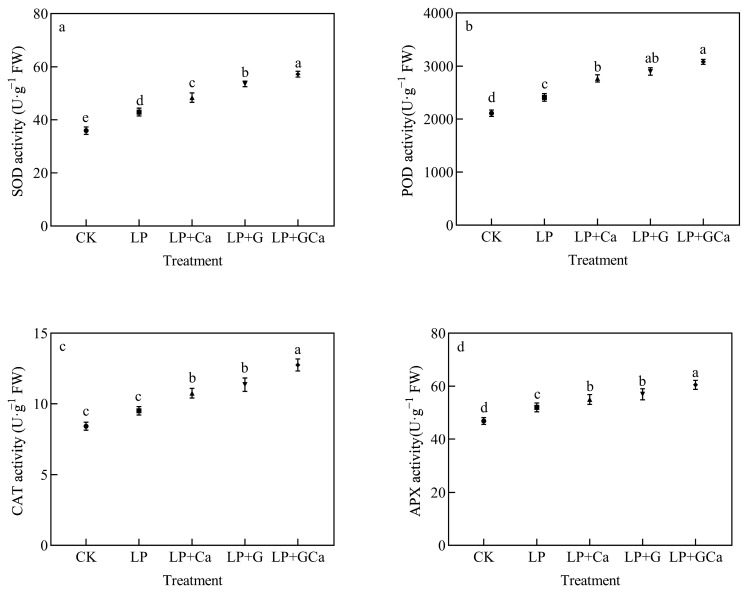
Effects of exogenous GABA-Ca on SOD (**a**), POD (**b**), CAT (**c**), and APX (**d**) activity in peanut leaves under low-P stress. Values are means of three biological replicates ± SE (*n* = 3). Different and unshared letters above columns in the figures indicate significant differences among the 5 variants (*p* ≤ 0.05).

Compared with CK, LP treatment induced higher antioxidant enzymatic activity in leaves. The application of GABA and Ca^2+^ significantly enhanced the antioxidant activity of the peanut plants except for control or CK plants (Figure 4a,b,d). The LP + GCa treatment showed a significant difference from all other remaining treatments. The LP + GCa treatment significantly enhanced SOD, POD, CAT, and APX enzyme activities (Figure 4a–d). However, the LP + G treatment showed higher antioxidant enzyme activities compared with LP + Ca.

### 3.5. Photosystem I and II Activity

**Figure 5:**
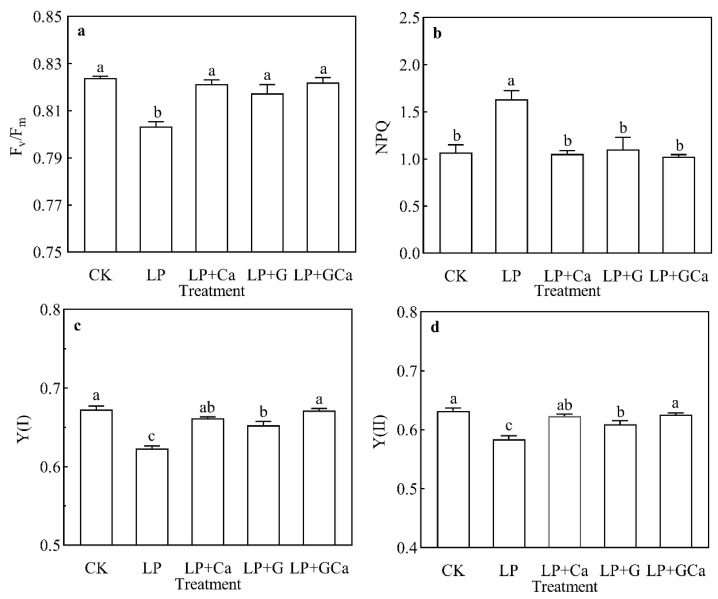
Effects of exogenous GABA-Ca on F_v_/F_m_ (**a**), NPQ (**b**), Y (I) (**c**), and Y (II) (**d**) in peanut leaves under low-P stress. Values are means of three biological replicates ± SE (*n* = 3). Different and unshared letters above columns in the figures indicate significant differences among the 5 variants (*p* ≤ 0.05).

Compared with CK, LP treatment was associated with lower Y (I), Y (II), and F_v_/F_m_ in leaves. Compared with LP, LP + Ca, LP + G, and LP + GCa treatments significantly increased the F_v_/F_m_ in peanut leaves under low-P stress (Figure 5a). Compared with LP, LP + Ca, LP + G, and LP + GCa treatments significantly decreased the NPQ in peanut leaves under low-P stress (Figure 5b). CK and LP + GCa had the highest Y (I), followed by LP + Ca and LP + GABA, with the lowest values observed in LP (Figure 5c). CK and LP + GCa treatments had the highest Y (II), followed by LP + Ca, while the lowest was observed in LP treatment (Figure 5d).

### 3.6. ETR and CEF

**Figure 6:**
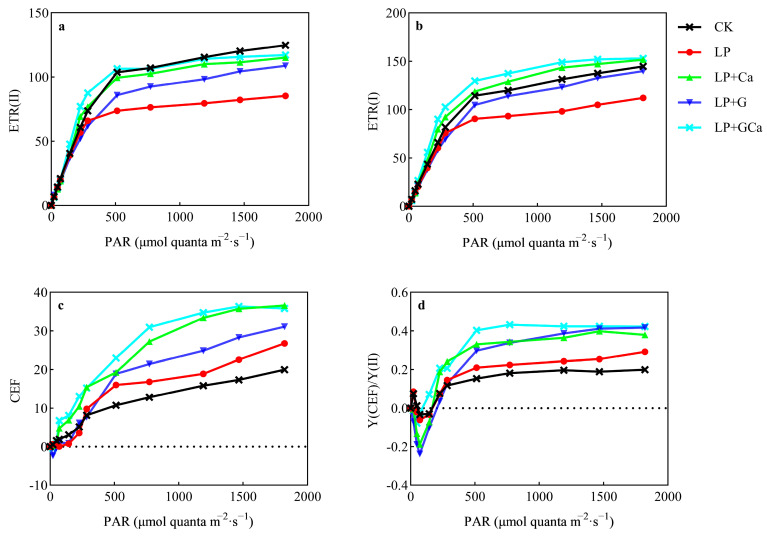
Effects of exogenous GABA-Ca on ETR (II) (**a**), ETR (I) (**b**), CEF (**c**), and Y (CEF)/Y (II) (**d**) in peanut leaves under low-P stress. Values are means of three biological replicates ± SE (*n* = 3).

The light response curve indicated the relative electron flow rate of the photosystem under different light intensities (Figure 6a,b). The difference between ETR (II) and ETR (I) was cyclic electron flow (Figure 6c). Compared with CK, LP treatment was associated with lower ETR (II) and ETR (I) in leaves (Figure 6a,b). LP + GCa treatment enhanced both ETR (II) and ETR (I) under high-light conditions compared with LP (Figure 6a,b). Compared with CK, LP treatment was associated with higher CEF in leaves (Figure 6c). LP + Ca was associated with higher ETR (II), ETR (I), and CEF compared with LP + G (Figure 6a–c).

### 3.7. ∆pH and ∆Ψ

**Figure 7:**
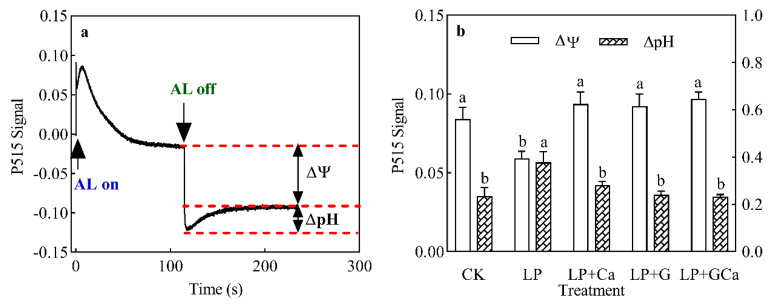
Effects of exogenous GABA-Ca on the proton gradient (∆pH) and membrane potential (∆Ψ) (**a**,**b**) in peanut leaves under low-P stress. Values are means of three biological replicates ± SE (*n* = 3). Different and unshared letters above columns in the figures indicate significant differences among the 5 variants (*p* ≤ 0.05).

The increased ∆pH of the thylakoid indicated thylakoid lumen acidification. Compared with CK, LP treatment was associated with a higher ∆pH of thylakoids in leaves (Figure 7a,b). Compared with LP treatment, LP + Ca, LP + G, and LP + GCa treatments significantly reduced the ∆pH of thylakoids in peanut leaves under low-P stress (Figure 7b).

### 3.8. Thylakoid Membrane Integrity and ATP Synthase Activity

**Figure 8:**
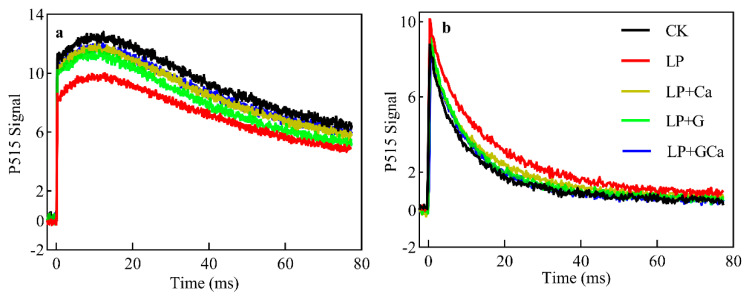
Effects of exogenous GABA-Ca on rapid P515 kinetics (**a**,**b**) in peanut leaves under low-P stress. Rapid kinetics over 1 h in darkness: thylakoid membrane integrity (**a**). Rapid kinetics under light exposure for 10 min at 1000 μmol photons·m^−2^·s^−1^ and darkness for 4 min: ATP synthase activity (**b**) in peanut leaves under low-P stress. Values are means of three biological replicates ± SE (*n* = 3).

The faster decay of the P515 signal in the dark indicated that the thylakoid membrane integrity was impaired (Figure 8a). The lower decay of the P515 signal after light exposure indicated that ATP synthase activity was inhibited (Figure 8b). Compared with CK, LP treatment was associated with lower thylakoid membrane integrity and ATP synthase activity in leaves (Figure 8a,b). LP + GCa treatment significantly enhanced thylakoid membrane integrity and ATP synthase activity (Figure 8a,b).

## 4. Discussion

Low availability of P in soils is one of the key factors affecting crop production [1], and the cultivation of peanuts is highly sensitive to P supply [12]. A search of the literature revealed that there are limited studies pertaining to the improvement of growth and physiological resilience to low-P conditions for peanuts. Guo et al. [30] and Wu et al. [42] demonstrated that exogenous GABA application was effective in improving plants’ resilience to a variety of abiotic stresses, and Sun et al. [13] recently confirmed this statement. Exogenous Ca^2+^ is also beneficial for mitigating low-P stress in peanuts [15]. Additionally, there may be complex signal crosstalk between GABA and Ca^2+^ [30]. At present, there is limited information regarding the effects of GABA-Ca on strengthening plants’ resilience to low-P conditions. Our study demonstrated that a combination of GABA-Ca, containing 10 mM GABA and 15 mM Ca^2+^, alleviated the sub-optimal physiological status of peanut plants growing in low-P conditions.

### 4.1. Effects of GABA-Ca on Growth, Leaf Nutrients, and ROS in Peanut Under Low-P Stress

Exogenous GABA-Ca enhanced the height (Figure 1a), dry matter weight (Figure 1b), and chlorophyll concentration (Figure 1c) of peanut plants under low-P stress and alleviated the influence of P deficiency on peanut growth. Previous studies have shown that GABA and Ca^2+^ restored the growth inhibition of peanuts under low-P stress [13,15]. This study further demonstrated that the combination of GABA and Ca was more effective (Figure 1) in enhancing P (Figure 2a), N (Figure 2b), K (Figure 2c), Ca (Figure 2d), Mg (Figure 2e), Fe (Figure 2f), Mn (Figure 2g), Cu (Figure 2h), and Zn (Figure 2i) concentrations in peanut leaves under low-P stress, while P deficiency lowered the N, P, K, Ca, and Mg concentrations in peanut leaves. GABA generally regulates the activity of plant-specific anion transporters and is also recognized as a plant ion regulator [43,44]. GABA delivered a better effect on mineral element accumulation in leaves than Ca under LP. However, Ca had a better effect on SPAD and plant dry weight than GABA under LP.

Based on our analyses, the O_2_^•−^ generation rate (Figure 3b), H_2_O_2_ content (Figure 3a), MDA content (Figure 3c), antioxidant enzyme activity (Figure 4), and thylakoid membrane integrity (Figure 8a) demonstrated that exogenous GABA-Ca alleviated the photooxidative stress induced in peanut leaves by low-P stress. P deficiency induces oxidative stress and damages cell membranes [13]. The application of GABA has been found to enhance the antioxidant enzyme activity of maize under salt stress and reduce the levels of O_2_·^−^ and MDA in leaves [45]. GABA also enhances the antioxidant capacity of seedlings under stress and significantly decreases ROS and MDA accumulation [46]. Exogenous GABA improved the activity of antioxidant enzymes such as POD, CAT, and SOD, and it diminished the steady-state level of ROS. Existing study results suggest that GABA application, which reduces the levels of accumulated O_2_·^−^, H_2_O_2_, and MDA, results in alleviation of oxidative damage in plants under drought and salt stress [47]. Similarly, Ca^2+^ alleviates oxidative damage in crops under stress by enhancing the activity of enzymes such as SOD and CAT, which are crucial for the detoxifying action of the ROS scavenging system in plants [48]. Sun et al. [15] found that exogenous Ca^2+^ reduces the ROS levels in peanut plants under low-P stress, thereby mitigating oxidative stress damage. GABA also delivered a better effect on antioxidant enzyme activities than Ca under LP. GABA and Ca^2+^ are closely involved in antioxidant defense mechanisms, and combined GABA-Ca treatment further enhances the antioxidant capacity of peanuts under low-P stress and enhances their resistance to low-P stress.

### 4.2. GABA-Ca Enhances CEF and Alleviates Photoinhibition in Peanut Leaves Under Low-P Stress

Exogenous GABA has been proven to regulate the photosynthetic electron transport chain (PETC) in plants by reducing the stress induced by ROS. The excess electrons in the PETC may increase ROS production, thereby reducing photosynthetic activity. Based on our analyses, exogenous GABA-Ca decreased the acidity of the thylakoid lumen (Figure 8a) and increased linear electron flow (LEF) (Figure 6a,b) and CEF (Figure 6c,d) in leaves of peanuts under low-P stress. The water splitting and plastoquinone (PQ) cycle within the PETC transports H^+^ into the thylakoid lumen [49]. Therefore, improvements in the CEF and LEF contribute to H^+^ accumulation in the thylakoid lumen [49]. Controlled H^+^ efflux via ATP synthase in the thylakoid lumen is a key factor in the photoprotection mechanism of “photosynthetic control” [50]. Under low-P stress, the activity of ATP synthase (Figure 8b) in the thylakoid of leaves decreased, leading to the accumulation of protons in the thylakoid lumen (Figure 7b). This regulates photosynthetic control mechanisms aligned with the previous findings [13,15]. Photosynthetic control mechanisms are crucial for the photosystem because they adjust photosynthetic electron transport to balance energy supply and demand [51]. The increase in ATP synthase activity is the main factor through which GABA-Ca alleviates the acidification of the thylakoid lumen in peanut leaves under low-P stress.

GABA-Ca enhanced the CEF in peanut leaves under low-P stress, particularly under high light intensity (Figure 6). Under LEF-limited conditions, CEF around PSI can contribute a higher ΔpH intensity [52]. Under low-P stress, an increase in the CEF helps to achieve photosynthetic control [13]. Both GABA and Ca enhance the CEF of peanuts under low-P stress and alleviate photoinhibition [13,15]. However, it appears paradoxical that CEF transports more H^+^ to the thylakoid lumen, where acidification needs to be reduced. CEF includes two pathways: proton gradient regulation 5 (PGR5)/PRG5-like photosynthetic phenotype 1 (PGRL1) and NADH dehydrogenase-like complex [53]. Lu et al. [54] showed that the inhibiting pathway of CEF significantly reduces the ΔpH in the thylakoid. The increase in CEF (Figure 6) induced by GABA-Ca under low P was not considered to conflict with the decrease in H^+^ (Figure 7). The significant increase in and flexible regulation of CEF induced by GABA-Ca contributed to maintaining ∆pH stability, prevent an excessive decrease in ΔpH due to substantial H^+^ efflux induced by ATP synthase activity contributing to the excessive acidification of thylakoid lumen. Improvement in CEF is necessary despite the generation of a ΔpH gradient without PSII [54]. Our results suggest that GABA-Ca-induced CEF elevation is beneficial for the regulation of thylakoid acidification.

## 5. Conclusions

Low-P stress in peanut plants induced severe leaf-level photoinhibition and ROS toxicity that significantly limited photosynthesis and growth. Exogenous GABA-Ca effectively improved the sub-optimal physiological status under low-P stress through various processes: it activated the antioxidant enzyme system, reduced oxidative damage to the photosynthetic membrane, and decreased the ROS toxicity induced by low P. Additionally, exogenous GABA-Ca effectively balanced the H^+^ accumulation in the thylakoid lumen by enhancing the CEF and increasing ATP synthase activity. Under low P, exogenous GABA restored key physiological attributes such as plant height, foliar mineral content, and antioxidant enzyme activities. Interestingly, exogenous Ca delivered better effects on foliar chlorophyll (SPAD values), plant dry weight, and photochemical activity than GABA. Compared to the sole application of either Ca^2+^ or GABA, combined GABA-Ca application further alleviated P deficiency symptoms, including increased root–shoot ratios, photoinhibition, and ROS toxicity. The underlying mechanism of GABA-Ca interactions in plants is unclear and requires further research. We postulated that there might be a plausible signal amplification between GABA and Ca^2+^, suggesting that the combination of GABA and Ca may offer an effective strategy for enhancing peanut adaptation to low-P conditions. Moving forward, the strategic supplementation of GABA-Ca, either during cultivation or by formulating novel fertilizers, opens up many possibilities for better and more resilient plant production.

## Data Availability

The data presented in this study are available on request from the corresponding author.
